# Development of Online Tool Wear-Out Detection System Using Silver–Polyester Thick Film Sensor for Low-Duty Cycle Machining Operations

**DOI:** 10.3390/s22218200

**Published:** 2022-10-26

**Authors:** Jegadeeshwaran Rakkiyannan, Lakshmipathi Jakkamputi, Mohanraj Thangamuthu, Abhishek D. Patange, Sakthivel Gnanasekaran

**Affiliations:** 1 Center for Automation, School of Mechanical Engineering, Vellore Institute of Technology, Chennai 600127, India; 2 School of Mechanical Engineering, Vellore Institute of Technology, Chennai 600127, India; 3 Department of Mechanical Engineering, Amrita School of Engineering, Amrita Vishwa Vidyapeetham, Coimbatore 641112, India; 4 Department of Mechanical Engineering, College of Engineering Pune, Pune 411005, India

**Keywords:** wear-out detection, thick film sensor, polyester substrate, single point cutting tool, low-duty cycle

## Abstract

This paper deals with the design and development of a silver–polyester thick film sensor and associated system for the wear-out detection of single-point cutting tools for low-duty cycle machining operations. Conventional means of wear-out detection use dynamometers, accelerometers, microphones, acoustic emission sensors, thermal infrared cameras, and machine vision systems that detect tool wear during the process. Direct measurements with optical instruments are accurate but affect the machining process. In this study, the use of a thick film sensor to detect wear-out for aa real-time low-duty machining operation was proposed to eliminate the limitations of the current methods. The proposed sensor monitors the tool condition accurately as the wear acts directly on the sensor, which makes the system simple and more reliable. The effect of tool temperature on the sensor during the machining operation was also studied to determine the displacement/deformation of tracing and the polymer substrate at different service temperatures. The proposed tool wear detection system with the silver–polyester thick film sensor mounted directly on the cutting tool tip proved to be highly capable of detecting the tool wear with good reliability.

## 1. Introduction

Tool wear-out detection systems are used on the production floor to determine the wear-out of the tool. It is essential to monitor the tool during operation as any wear-out or breakage will affect the product quality and increase the production time and cost. Traditional tool wear-out detection methods include profilometry and optical and radiographic methods [1]. There are two tool wear systems today: the contact type and non-contact type. The contact type includes sensors in direct contact with the tool.

In contrast, non-contact sensors indirectly measure related physical quantities that change with the machining process and establish a realistic mathematical model between the relevant physical quantities and tool wear [2]. These conventional measurements use vibration, acoustic, visual, and thermal methods to detect tool wear [3,4]. The measurement is based on indirect means such as vibrations signals from an accelerometer [5], acoustic emission (AE) [6,7], sound sensors [8], and infrared imaging for thermal optics and infrared thermometers [9].

AE might be adequately available in the complete machining zone; selecting a suitable location to fix the AE sensor to grab appropriate AE signals is questionable, as insight into the AE path has to be determined. A disadvantage of employing AE as an indicator of tool wear is that its signals are more susceptible to machining and sound variations than the tool condition itself. Utilizing AE as an individual sensor to observe the cutting tool condition is challenging [4]. The static and dynamic components of the cutting force have a significant effect on tool wear. The measurement of cutting force dynamics requires piezo-electric dynamometers, which are highly expensive and not affordable by small and medium-scale industries [10]. The correlation between vibration signals and the cutting forces establishes the dynamic nature of the material removal process. Using cutting force and vibration sensors increases the signal processing complexity and cost [11].

The tool condition is monitored using the cutting zone temperature. The cutting temperature increases with speed and feed [12]. The cutting temperature can be decreased using coolant [13] and the cryogenic cooling technique [14], and the cutting tool performance can be increased by machining with appropriate cutting speeds [15]. The analytical model can calculate the cutting tool temperature [16], and the high-speed machining of more rigid materials increases the tool temperature and decreases the tool life [17]. The friction model can also predict the chip form, cutting force, and temperature [18]. It is possible to analyze the cutting tool and workpiece from the chip temperature approximately, which leads to an error in obtaining the exact value. It becomes a disadvantage when working in high temperatures [11].

A vision system with a complex image processing algorithm is essential for tool wear monitoring, which is a challenging task [17]. The indirect measurement methods are often costly compared to other modes of wear detection. Hence, a direct measurement is required for tool wear-out detection. Thin/thick film sensors are material traces deposited over a film or substrate. These can be used as surface coatings or as individual components for a wide range of sensory applications at a minimum cost. Another benefit of using a thin/thick film sensor for wear measurement is its simple installation on any surface or system with a minimum effect on the measurement operation. A hydrogenated carbon layer-based thin film sensor was developed for examining ball screw drives. They reported improved reliability of the sensor in determining the condition of the ball screws [19]. A tool-integrated thin-film sensor with four-layers on the surface of cemented carbide cutting inserts was developed to measure the temperature of the cutting tool and tool wear [20].

A thin film transducer with an Al_2_O_3_ base coating was used to detect the tool wear. The effect on the sensor due to thermal stresses was explored. Wear can also be measured using resistance materials. When materials wear-out, there is a corresponding change in resistance, which can be used to detect the wear-out [21]. The resistance-based approach is limited due to the variation in resistance with temperature. The tool heated up after a specific operation time will negatively affect the sensor accuracy. The necessary properties of the thick film sensor tracer material include good electrical conductivity, adhesion to the shielding layers, and minimal ductility. Aluminum oxide and tantalum oxide were suitable materials for thick film sensor tracing. However, the chemical vapor deposition method used in the fabrication proved to be a real challenge. Indium–tin oxide was also found to have high conductivity, but the fabrication cost was high [22].

The TiAlN coating on the tool flank face was used for tool temperature measurement during operation [23]. The vapor deposition of thin film sensors on the flat and curved surface was performed to detect flank wear land width [24]. A strain gauge sensor was integrated into the tool to measure forces during machining operations [25]. An MXene thin film sensor was used to monitor the health condition of the repaired composite structure [26]. The MXene material has high conductivity, specific surface area, excellent hydrophilicity, exceptional mechanical properties, and other characteristics. MXene, combined with appropriate materials, is used to design strain and temperature sensors for health monitoring applications [27]. Wearable physical sensors made from graphene are used to develop strain gauge, temperature and pressure sensors for health monitoring, human–robotic interface, and therapeutics [28]. A reduced graphene oxide (r-GO) based flexible strain gauge sensor was fabricated and reproduced the characteristics of the strain gauge [29].

A wear-resistant thin film sensor was developed using Al_2_O_3_ and Cr to measure the temperature during the turning process. It was found that the sensor resistance gradually increased with an increase in temperature. The fabricated sensor was tested by turning AISI 4141 steel. The delamination was found in the SEM images when the tool became worn-out. The delamination can be avoided by providing enhanced adhesion between the layers [30]. Thin film sensors (thickness of a few μm) with polymer substrates cannot be used for wear detection applications such as in cutting tools as they cannot withstand moderate cutting tool temperatures occurring during operation. Therefore, a thick film sensor (thickness range of 50–100 μm) can be used for a particular application with the same principles involved in thin film sensor operation. The main limitation of using thin/thick film sensors on tools is the temperature generated during heavy-duty cycle machining operations, which affect polymer sensors.

The increased temperature will also affect the tracing–substrate contact due to variations in the coefficient of thermal expansion of the two materials [31]. The thermal expansion of a material is caused by the increase in the average inter-atomic distance during heating. The problem of inter-atomic distance can be avoided by using the system only for low-duty cycle machining operations or high-temperature-resistant polymers. The silver–polyester thick film sensor and associated wear detection system advocated in this study used the conductance method to monitor the wear by attaching the film sensor to the tooltip.

From the literature, it was observed that there was no trace of research on thick film sensors to detect wear in single-point cutting tools. Furthermore, as in resistance-based wear detection methods, few attempts have been made to fabricate and test a wear sensor with a direct mode of operation (wear acting directly on the sensor). Therefore, an attempt was made to design, fabricate, and test a thick film sensor positioned at the tooltip and related wear detection system to measure the wear with high accuracy and reliability. Aside from wear detection, sensor temperature monitoring during low-duty cycle operation, and thermal displacement analysis of the proposed thick film sensor materials were also performed. The proposed methodology includes thick film sensor design, analysis, fabrication, wear detection system design, and component assembly, followed by lathe testing for sensor temperature and tool wear.

## 2. Design, Fabrication, and Analysis

The design phase includes selecting suitable materials for the substrate and tracing thick film to fulfill the required application. Integration with the tool wear detection system also needs to be considered so that the fabricated sensor can respond to the system reliably and efficiently.

### 2.1. Materials

Material selection is an essential feature of designing a thick film sensor. It generally depends on the application, operating environment, and costs involved. The sensor material must satisfy the electrical and mechanical functional requirements. The various materials suggested by the literature are given in Table 1 [21]. From Table 1, polyester was chosen as the substrate material because its electrical resistivity, melting point, and hardness properties allow for immediate tool wear to be felt. This material reduces the error value detected by cutting tools.

#### 2.1.1. Substrate Material

The substrate material for the tracing deposition must be inexpensive and have good chemical inertness. Though standard polymers fulfill the basic needs, the temperature acting on the sensor due to tool heating is a significant constraint. Regular polymers have shallow melting points and quickly melt off once the tooltip is heated. Polyester was selected because of the quality of the material. The tracing material adherence on polyester is also good, and it is generally used to make flexible circuit sheets in the electronics industry. The polyester properties are depicted in Table 2.

#### 2.1.2. Tracing Material

Aluminum was first considered, but its deposition on a polymer film was a constraint. A nickel/iron alloy was also considered, but the increased cost prohibited its use. Silver ink, which is most commonly used in the electronics industry [32], was the optimum choice due to its easy availability and everyday usage in making circuit sheets. Furthermore, the metal is inert and a good conductor. Depositing the material on a polymer film is also very easy due to its suitable adhesive property.

### 2.2. Sensor Design

**Figure 1:**
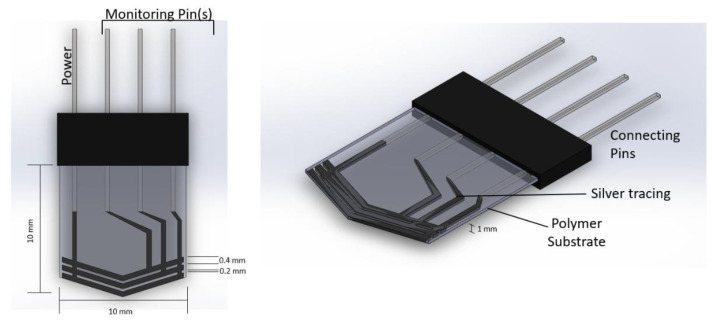
Sensor design and its dimensions.

Wear rate detection is based on the conductivity of the coated material on the polymer film. The tracing material must be able to pass a constant current through the tracings for proper measurement by the detection system at the receiving end. Figure 1 shows the designed sensor and its dimensions. The coating width of conducting lines is 0.4 mm, and the gap width is 0.2 mm. The range of wear detection for the designed sensor is 1.4 mm to 2.6 mm (Span: 1.2 mm). The range depends on the coating width of the tracing and sensor clearance from the tooltip, hence customizable.

An insulator layer made of mica of a 0.5 mm thickness was placed at one side to prevent the temperature from the tool affecting the polymer film. The sensor was fabricated according to the geometric tool profile to be placed on the tooltip effectively.

### 2.3. Sensor Fabrication

**Figure 2:**
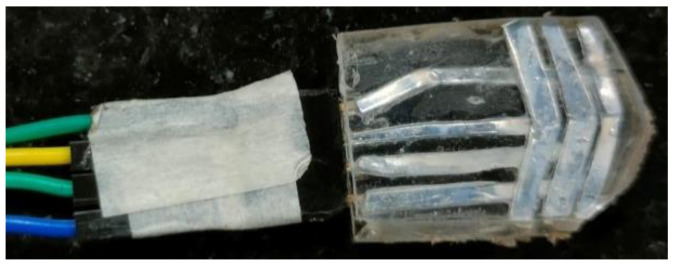
Fabricated sensor.

A thick film sensor was fabricated using the heat lamination method. The tracing pattern was first printed to make the stencil. It was then placed on the substrate, and tracer ink was poured. A squeegee was used to apply pressure to the ink to cover over stencil. The ink passed through the pattern shape onto the substrate, where it was deposited and dried. The printed sensor was cured at 120 °C for 20–30 min. The pattern shape on the stencil could be easily altered according to the tool/tooltip geometric profile. The polymer thickness was 60 μm, and the metal tracing thickness was 20 μm. A silver–polyester thick film sensor of 10 mm × 10 mm × 1 mm was fabricated using this method, as shown in Figure 2.

### 2.4. Thick Film Sensor Analysis

A silver-based thick film sensor was analyzed for the thermal expansion of constituent metal tracing. The continuous operation will cause the tool to become heated. Hence, it needs to be analyzed for thermal expansion and electrical conductivity at a maximum serviceable temperature to ensure its proper working.

#### 2.4.1. Thermal Expansion of Metal Tracing

The thermal expansion of silver tracing with a length of 10 mm and 0.4 mm thickness is given by Equation (1).

(1) $$A=A_{0}\left(1+2\alpha ∆T\right)$$

where *A* is the final area after thermal expansion; *A*_0_ is the initial area of tracing; *α* is the coefficient of thermal expansion (for silver *α* = 19.5 × 10^−6^ m/mK); and Δ*T* is the increase in temperature.

Theoretical results for the thermal expansion of silver tracing at different temperatures are shown in Table 3. The results indicate that thermal expansion was minimal at low to moderate temperatures, but became significant at higher temperatures.

The Wiedemann–Franz Law states that the thermal conductivity of silver tracing to bulk silver is proportional to the electrical conductivity of silver tracing to bulk silver [33]. Thus, any temperature variation will affect the tracing’s electrical conductivity, which is given by Equation (2).

(2) $$K_{tracing}/\sigma_{tracing}=K_{bulk}/\sigma_{bulk}=LT$$

where *K_tracing_* is the thermal conductivity of silver tracing; *σ_tracing_* is the electrical conductivity of silver tracing; *K_bulk_* is the thermal conductivity of silver bulk; σ*_bulk_* is the electrical conductivity of silver bulk; L is the Lorenz number, and *T* is temperature.

The maximum service temperature for the polymer substrate is 150 °C (423 K). Assuming thermal conductivity to be a constant 406 W/mK, the Lorenz number is 2.32 × 10^−8^ [34], and the σtracing will be 4.1 × 10^7^/Wm. It was observed that the electrical conductivity of the silver tracing was not severely affected at the maximum service temperature.

#### 2.4.2. Thermal Deformation Analysis

Tracing and substrate were modeled and analyzed separately to study the effects of temperature on the thick film sensor.

##### Tracing

**Figure 3:**
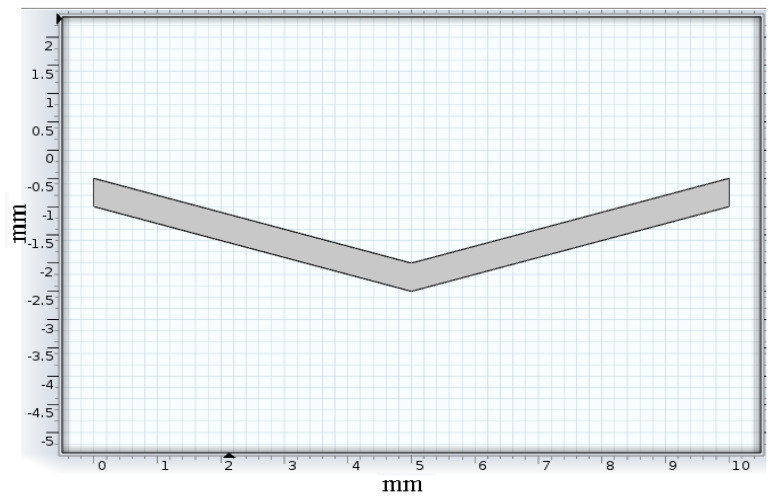
Tracing model.

Silver tracing on the thick polymer film was modeled (Figure 3) and analyzed using Solidworks design and simulation software for displacement occurring on it at two different temperatures. The initial tracing temperature was taken as 300 K (27 °C). The base of the 3D tracing model was constrained to simulate the constraint caused by the substrate material deposited on the tracing material. The maximum service temperature of the silver–polyester thick film sensor was 423 K (150 °C), after which the polyester substrate will be damaged. Temperatures of 323 K (50 °C) and 423 K (150 °C) were applied on the base to study the effect of temperature. The temperature rise was gradual as heat formation on the tool depends upon the duration of operation, feed, and depth of cut.

##### Tracing Substrate

**Figure 4:**
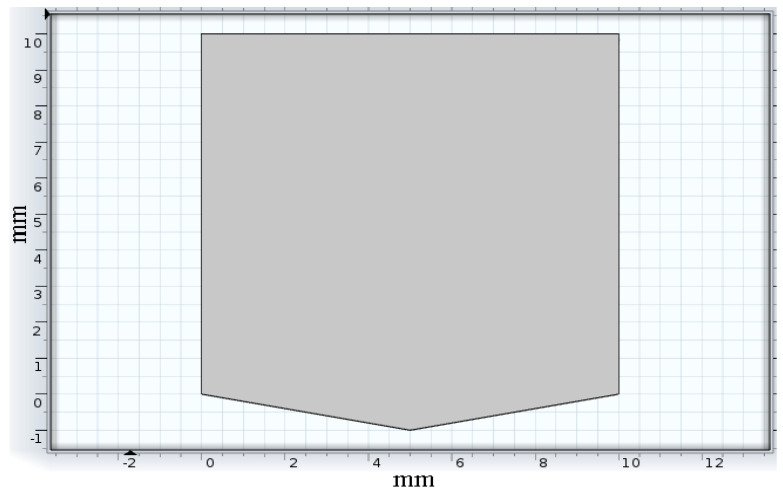
Substrate model.

The polyester substrate was modeled (Figure 4) and analyzed for thermal displacement. The base was constrained to simulate the fixed positioning of the substrate on the insulation layer. A temperature load of 323 K (50 °C), the low duty cycle sensor temperature, and 423 K (150 °C) maximum service temperature were applied at the base for displacement study of the substrate due to temperature.

## 3. Tool Wear-Out Detection System Design and Operation

### 3.1. Design

The tool wear detection system used a high-performance, low-power RISC-based 8-bit microcontroller unit (MCU) for its operation. The proposed system comprised a temperature sensor (LM 35), thin-film sensor, liquid crystal display (LCD), and power supply unit. LM35 is a 3-terminal temperature sensor that measures the surrounding temperatures ranging from −55 °C to 150 °C. It does not require any external calibration circuitry. The sensor has a sensitivity of 10 mV/°C, an accuracy of 0.5 °C, an operating range of −55 °C to 155 °C with a 4–30 V operating voltage, and a low impedance output of 0.1 Ω for a 1 mA load. As the temperature increases, the output voltage also increases. The temperature sensor (LM 35) as connected to the microcontroller’s ADC0 port (pin 40). A thin film sensor was used to detect the wear-out condition of the cutting tool. The thin film sensor consisted of four pins. A typical power line of +5 V joined all the lines and was connected with the first pin. The other ends of the three lines were connected to the microcontroller’s interrupt (INT0, INT1, and INT2—pins 16, 17, 3, respectively) pins.

**Figure 5:**
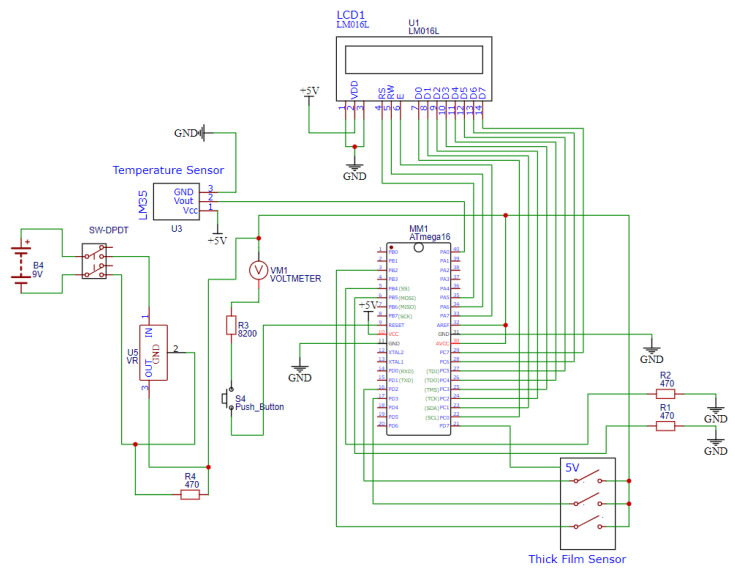
Tool wear-out detection system circuit.

The current was constantly monitored at the microcontroller end. Breakage of the tracing lines will lead to interruption in the generation and can be detected. During machining, the wear sensor detected the three wear levels (1.4 mm, 2 mm, and 2.6 mm) with a span of 1.2 mm. The LCD was connected to port C and used to display the tool temperature and tool condition. The tool condition and sensor temperature are shown on the LCD screen. Figure 5 shows the circuit used to test the thick film sensor and monitor the tool condition.

### 3.2. System Operation

**Figure 6:**
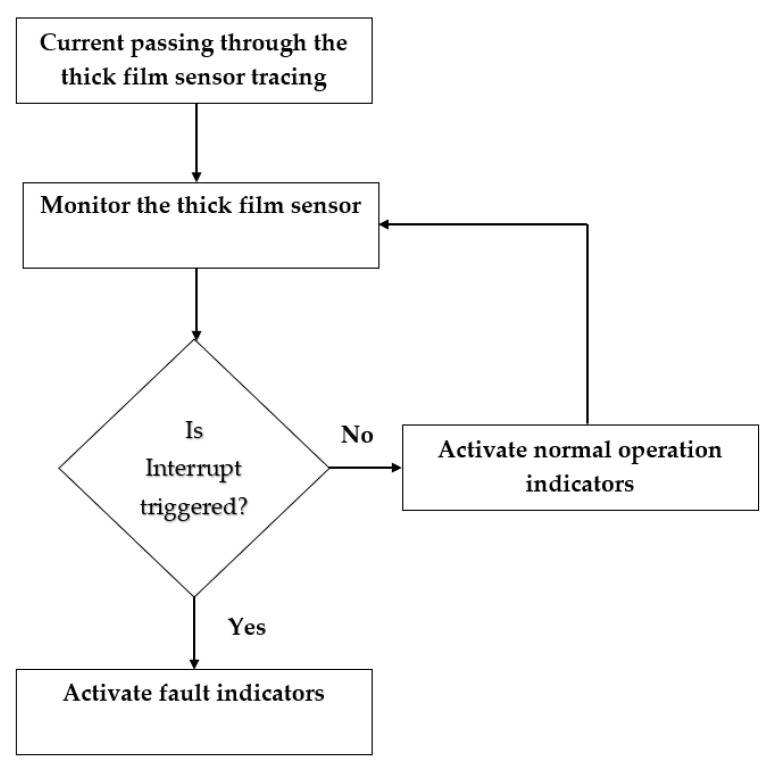
Flowchart for the system operation.

The operation of the thick film sensor and wear-out detection system are shown in Figure 6. The first pin of the thick film sensor transmitted power (+5 V) through the silver tracings on the polymer substrate. The second, third, and fourth pins were connected to the interrupt pins of the microcontroller. The controller keeps checking the HIGH condition of the pins. If the power supply flows through all of the pins, the output is HIGH, and the LCD shows the tool’s normal condition. The LCD displays an alert message if a power fall in any of the pins is detected (LOW). Apart from the wear-out state, the sensor temperature is also displayed on the screen.

## 4. Sensor Integration and Testing

**Figure 7:**
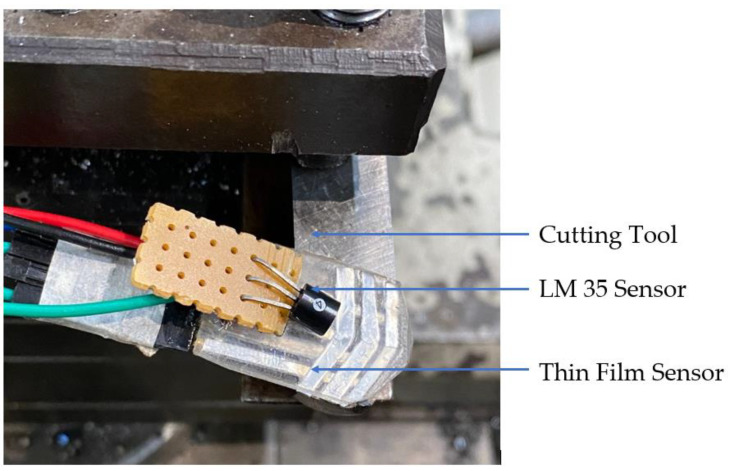
Thick film sensor and LM35 sensor at the tooltip.

**Figure 8:**
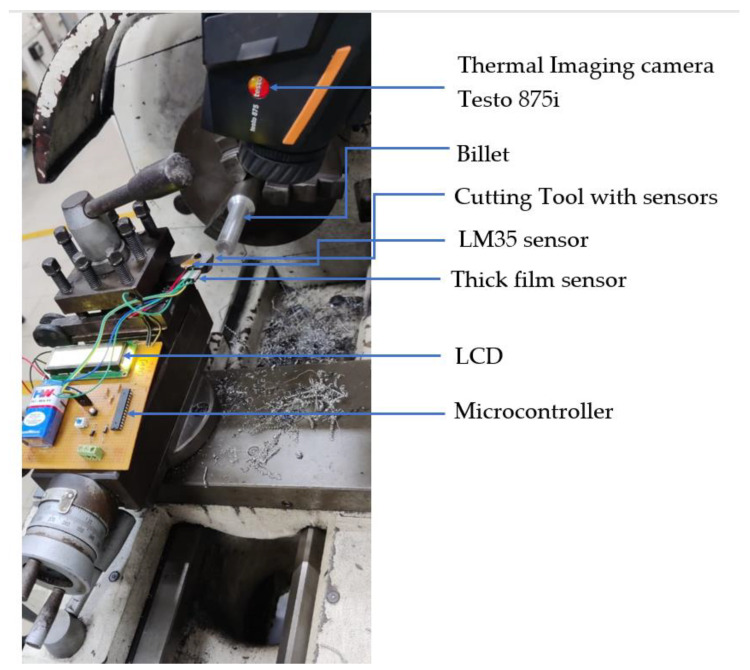
Cutting tool wear detection setup.

A silver-polyester-based thick film sensor was mounted on the sensor side of the insulating layer. The tracing was placed close to the tooltip at a clearance of 1 mm, as shown in Figure 7. Testing was carried out with a single-point cutting tool in a lathe machine, as shown in Figure 8. First, temperature readings from the thermal sensor placed on the mica insulator were used to monitor the temperature at the sensor end of the insulator layer adhered to the tool. The temperature measurement was performed to ensure that the temperature at the sensor end would be within the operating range of the thick film sensor.

The cutting tool used for turning was High-Speed Steels (HSS 18:4:1) containing 18% tungsten, 4% chromium, 1% vanadium, 0.8% carbon, and the remainder was iron. Mild steel with a billet size of 100 mm in length and 25 mm in diameter was positioned for turning operation. The material properties of mild steel are given in Table 4. The turning parameters used in this operation are given in Table 5. Cutting fluid usage was avoided to detect the proper temperature rise at the insulating layer’s sensor side to study the sensor temperature’s dependence on the operating time. The cutting depth was varied, and the temperature was recorded.

Additionally, the temperature was measured with a thermal imaging camera Testo 875i at the end of each pass to measure the cutting zone temperature. The thick film sensor outlets were connected to the wear detection system placed on the tool post, as shown in Figure 8. The tooltip was separately ground to simulate tip wear, and the tool wear detection system’s responses were noted for tool wear every 0.4 mm.

## 5. Results and Discussion

### 5.1. Results of Deformation Analysis

#### 5.1.1. Silver Tracing

**Figure 9:**
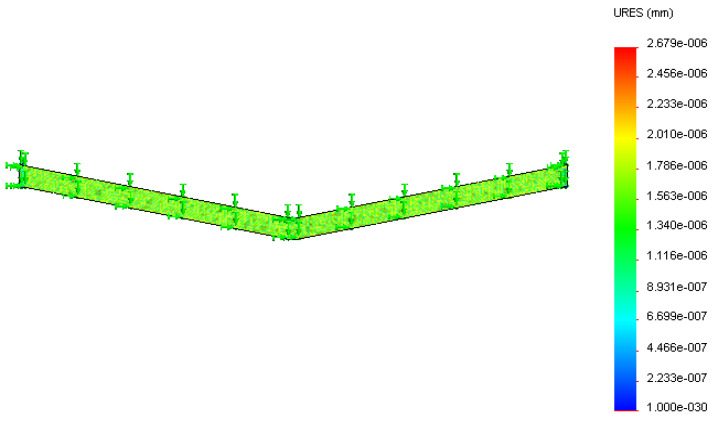
Displacement (in mm) of the silver tracing at 323 K.

**Figure 10:**
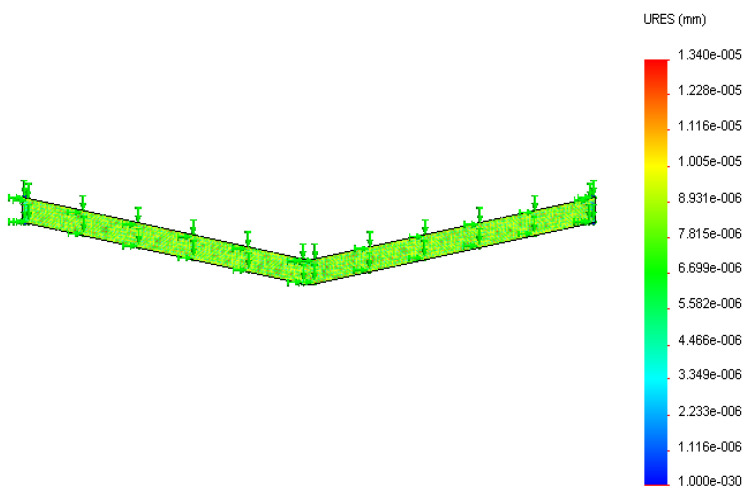
Displacement (in mm) of the silver tracing at 423 K.

The thermal analysis of tracing was conducted at 323 K (50 °C) and 423 K (150 °C). The tracing analysis shown in Figure 9 and Figure 10 depicts the displacement caused by the temperature at different surface regions of the silver ink tracing on the polymer. The maximum displacement occurring on the silver tracing was 2.679 × 10^−6^ mm at 323 K and 1.34 × 10^−5^ mm at 423 K. The results of the analysis of silver tracing depicts the almost even displacement of the tracing throughout the surface. This was due to the good thermal conductivity of silver, which evened out the temperature conducted at its base from the substrate.

#### 5.1.2. Polyester Substrate

**Figure 11:**
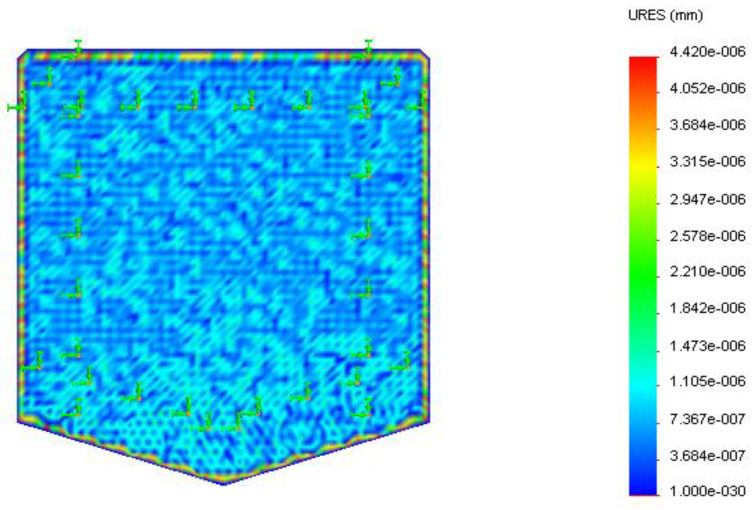
Displacement (in mm) of the polyester substrate at 323 K.

**Figure 12:**
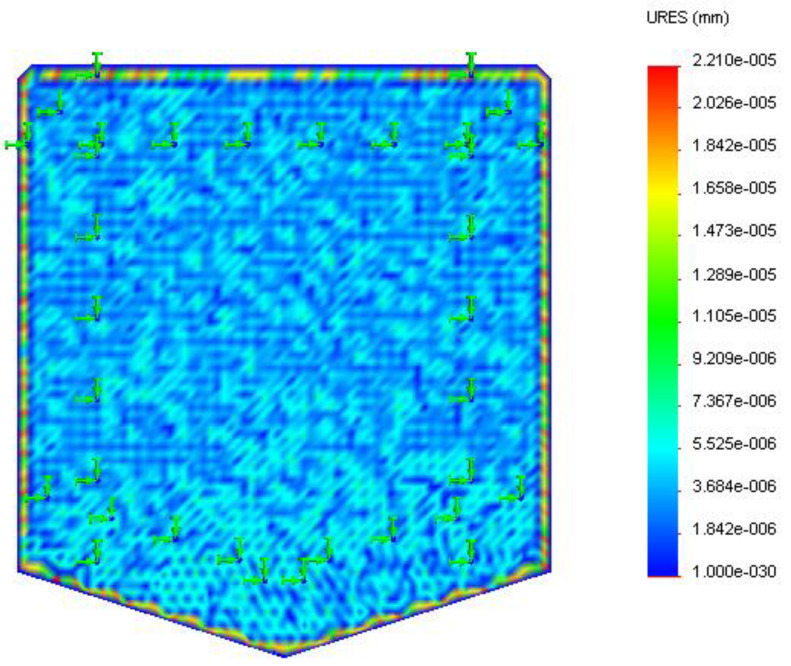
Displacement (in mm) of the polyester substrate at 423 K.

Thermal analysis of the substrate, as shown in Figure 11 and Figure 12, depicted the displacement caused by the temperature at different surface regions on the polyester substrate followed by the insulator layer. The maximum displacement of the substrate was 4.39 × 10^−6^ mm at 323 K and 4.42 × 10^−6^ mm at 423 K, respectively. It was observed that the maximum displacement occurred at the boundary regions as they were close to the hot surface compared to the bottom surface adhered on top of the insulator layer. Surface regions showed low displacement due to decreased thermal conductance by the polymer film from the base to its surface.

The thermal analysis results for maximum displacement are tabulated in Table 6 for the tracing and substrate. The results indicate that the displacement caused on the sensor by the temperature variation was significantly less. It could operate without any severe deformation at the specified temperature loads. The low displacement also indicated that the tracing–substrate contact was unaffected mainly at low temperatures, but stresses will be formed as more heat is generated during prolonged machining time.

### 5.2. Experimental Results

A temperature sensor connected to the wear detection system was used to record the sensor side temperature of the insulator film during low-duty cycle operation. Results revealed that the sensor temperature variation depends upon the depth of the cut and operation time. The sensor temperature was optimal for the polymer film operation in low-duty cycle operations. A gradual increase in the sensor temperature was observed with increased machining time and the depth of cut in the absence of a coolant fluid. Table 7 shows the temperature measured from the LM35 sensor (thick film sensor temperature) and thermal imaging camera Testo 875i (cutting zone temperature). The placement of the LM35 sensor was not closer to the cutting edge, hence the measured temperature value was lower compared to the cutting zone temperature. The thick film sensor temperature measurement ensured the safe working region of the sensor.

### 5.3. Tool Wear-Out Detection

The microcontroller accurately detected the tool wear for every 0.4 mm distance due to the degradation of silver tracing on the thick film. The tool degradation detection also depended on the thickness of the metal tracing (0.4 mm here) and a sensor clearance of about 1 mm. A small clearance has to be provided to prevent the sensor’s premature wear during regular operation. The clearance can be avoided by replacing the polymer substrate with a metal with similar material properties to that of the cutting tool. The sensor was modified to detect further wear by connecting the tracings to the remaining pins of the microcontroller.

Data from the microcontroller were sent to the cloud and could be monitored in real-time on a single screen. An infrared thermometer measured the temperature rise in the PET film and recorded the exact temperature of the film. This method is fundamentally different from conventional contact-based temperature measurement systems. The temperature sensor data were sent to a microcontroller, and the necessary conditioned data were pushed to the cloud and displayed on the screen.

### 5.4. Discussion

The thick film sensor was coated with a polymer film of silver using the heat lamination method because this coating method has a better life period when compared to other coating processes [35]. This coating process works better with a less volatile, safer solvent such as water, which is easily absorbed by the process. Compared to other deposition methods such as spray pyrolysis, sol–gel, dip coating, ultrasonic deposition, photochemical deposition, pulsed laser deposition, and spin coating [36], the used method has the following advantages such as a significantly reduced amount of coating applied, minimal weight gain, increased process efficiency and yield, and significantly reduced processing time due to suitable film-forming polymers and improved formulation flexibility [37].

The designed sensor was analyzed for thermal and displacement. The silver tracing and substrate analysis revealed that the displacement and temperature were in the sensor’s working range. This ensures the safe working of the sensors. During machining, the temperature was measured with LM35 and the thermal imaging camera Testo 875i. This revealed that the measuring accuracy of LM35 was in the acceptable range. The wear sensor detected the three wear levels with a span of 1.2 mm. When the tool is in working condition, the tracing will be there, and the interrupt pin of the microcontroller is not activated. This is indicated by the “normal” alert message displayed on the LCD. When the tool wear reached 1.4 mm, the first tracing line started to wear and produced the interrupt signals. The system displayed the “LOW” alert message. When the wear value exceeded 2 mm, the third tracing produced an interrupt signal, and the system displayed the message as “SEVERE”.

Whenever an alert message was received, the machining process was stopped, and the tool wear was measured manually to ensure reliable operation. The thick film sensor measures the tool wear rate depending upon the tracing dimensions. Table 8 shows the results obtained from the tool wear detection system. The sensor was tested on a single-point cutting tool and had an outstanding accuracy with a ±0.02 mm error value. The designed system can be effectively implemented in the turning process to monitor the tool wear levels. Furthermore, the tool conditions can be accessed by increasing the number of traces and the width of the tracing.

## 6. Conclusions

A thick film sensor and associated tool wear detection system were designed, fabricated, and tested. The new sensor proved accurate tool wear detection in the turning process with an error of ±0.02 mm. The new thick film sensor developed for the system can be improved to fit the tooltip profile better. Furthermore, it needs to be adopted as a rotating tool for drilling/milling operations. Additionally, a reduction in the thick film sensor size will also lead to enhanced results due to increased accuracy in wear measurement by a conducting coating of reduced size. The cost and size can be further reduced by integrating the entire system on a single chip. The sensor substrate can be replaced with a polymer with a high melting point for high tool temperature operation. It can control the machining process to produce dimensionally accurate parts with the desired surface quality. The novel sensor and associated system can also be used in other applications such as in the wear detection of bearings and brakes with few modifications.

## Figures and Tables

**Table 1:** The selection of an appropriate material for the substrate.

Material	*ρ* (Ω cm)	Hardness (kg/mm^2^)	Melting Point (°C)
AlN	10^9^	1225	2299
Al_2_O_3_	10^14^	2100	2047
Si_3_N_4_	10^12^	2200	1871
TiO_2_	10^14^	1100	1867
TiN	25 × 10^−6^	2100	2950
TiC	52 × 10^−6^	2800	3067
WC	17 × 10^−6^	2350	2776
Polyester	10^12^–10^14^	Barcol Hardness 68B	260

**Table 2:** The polyester substrate properties.

Property	Value
Density	1.39 (g/cc)
Modulus	5.1 (GPa)
Melting point	260 (°C)
Specific heat	0.28 (cal/g/°C)
Thermal expansion	1.7 × 10^−5^ (in/in°C)

**Table 3:** The thermal expansion of silver tracing.

Temperature (°C)	Thermal Expansion (mm^2^)
100	0.0156
150	0.0234
200	0.0312
250	0.039
300	0.046
350	0.054
400	0.062

**Table 4:** Properties of mild steel.

Properties	Values
Ultimate tensile strength	439.88 MPa
Yield strength	370.248 MPa
Elongation	15.0%
Rockwell hardness	B71

**Table 5:** Process parameters.

Parameters	Values
Cutting speed	100 m/min
Feed rate	0.2 mm/rev
Cutting depth	0.2–1.0 mm

**Table 6:** Maximum displacement in the deformation analysis.

Temperature	Silver Tracing	Polyester Substrate
323 K (50 °C)	2.679 × 10^−6^ mm	4.39 × 10^−6^ mm
423 K (150 °C)	1.34 × 10^−5^ mm	4.42 × 10^−6^ mm

**Table 7:** Temperature measurement results.

Depth of Cut (mm)	Thick Film Sensor Temperature—LM35 (°C)	Cutting Temperature—Thermal Imaging Camera Testo 875i (°C)
0.2	62.8	63.5
0.4	66.2	65.8
0.6	70.8	71.2
0.8	90.1	90.5
1.0	100.5	101.00

**Table 8:** Experiment results of the tool wear-out detection system.

S. No.	No. of INT Pins Enabled	Actual Tool Wear	Output from the Designed System
1.	0	1.30 mm	Normal
2.	1	1.4 mm	Level 1 wear/LOW
3.	2	2.0 mm	Level 2 wear/MODERATE
4.	3	2.3 mm	Level 3 wear/SEVERE

## Data Availability

Not applicable.
